# Assessing Task-Shifting Progress in Obstetrics and Gynecology: A Nationwide Survey of Japanese Obstetricians and Gynecologists

**DOI:** 10.3390/healthcare13080851

**Published:** 2025-04-08

**Authors:** Masatoshi Ishikawa, Ryoma Seto, Michiko Oguro, Yoshino Sato

**Affiliations:** Research Institute, Tokyo Healthcare University, Tokyo 140-0022, Japan; r-seto@thcu.ac.jp (R.S.); m-oguro@thcu.ac.jp (M.O.); 10yoshinosato05@gmail.com (Y.S.)

**Keywords:** task shifting, obstetrics and gynecology, physician workload

## Abstract

**Background/Objectives:** This study identified tasks suitable for delegation and gathered insights on how task shifting might affect medical care quality and working hours. **Methods:** A questionnaire survey was conducted among obstetricians and gynecologists working in hospitals nationwide. A multivariate logistic regression analysis was conducted. Then, opinions were collected on the individual tasks that should be task-shifted and the impact that task-shifting promotion would have on medical care quality and working hours. **Results:** Valid responses were obtained from 1164 doctors (16.3% of the 7127 obstetricians and gynecologists) working in hospitals. An analysis of the characteristics of the 31.2% of doctors who thought that task shifting had hardly progressed at their workplace showed that the odds of working 60–80 h were significantly higher (1.72, 95% CI: 1.06–2.77, *p* = 0.03) than those working <40 h and 3.50 (95% CI: 1.19–10.25, *p* = 0.02) for those working ≥100 h. Most obstetricians said that the only items “transferred” were “moving patients (e.g., from the operating room to the ward)”, “collecting blood culture samples”, and “ensuring chemotherapy lines”, revealing that task shifting had not progressed. Regarding the impact on medical care quality, if obstetricians and gynecologists promoted task shifting, most doctors said that the quality of care would improve, while 13% said that it would decrease. **Conclusions:** This study provides a comprehensive assessment of task shifting among OBGYNs in Japan, revealing its limited progress despite physician work reform efforts. Findings indicate that expanding task shifting could improve efficiency without compromising medical care quality, reinforcing its potential as a strategy for reducing physician workload.

## 1. Introduction

According to the World Health Organization (WHO), task shifting “presents a viable solution for improving healthcare coverage by making more efficient use of the human resources already available and by quickly increasing capacity while training and retention programs are expanded” [1].

Task-shifting initiatives to enhance medical efficiency are primarily promoted in all countries and have proved effective in various medical fields [2], including non-communicable diseases [3], HIV or AIDS [4], distribution of contraceptives [5], and the provision of primary care [6]. In 2012, the WHO released guidelines advocating for task shifting in maternal and newborn healthcare [7]. These results indicate that task shifting is a successful strategy for efficiently utilizing limited medical resources [7]. The Guidance Panel made 119 recommendations: 36 for lay health workers (LHWs), 23 for auxiliary nurses (ANs), 17 for auxiliary nurse midwives (ANMs), 13 for nurses, 13 for midwives, 8 for associate clinicians, 8 for advanced-level associate clinicians, and 1 for non-specialist doctors. The Guidance Panel excluded one priority question related to the distribution of misoprostol by any cadre to women during pregnancy for self-administration after childbirth.

In Japan, working hours are relatively long by international standards [8]. Physicians, in particular, have extensive working hours, with 42% of full-time physicians working over 60 h per week for more than 200 days a year—this rate is significantly higher than the average of 14% across all occupations [9]. Among physicians, those in obstetrics and gynecology (OBGYNs) have the longest working hours, with 20.5% working over 1920 h of overtime annually (averaging over 80 h per week) [10].

In 2016, the Ministry of Health, Labour and Welfare formed the Study Group on Physicians’ Working Style Reform to examine strategies for improving physicians’ work conditions. In 2018, the ministry introduced the Urgent Measures for Reducing Physicians’ Working Hours [11]. These measures emphasized strict oversight of working hours, leveraging existing occupational health systems, and encouraging task shifting as a means to decrease physicians’ workloads.

Specific methods for promoting task shifting included delegating tasks, such as preliminary examinations at the time of initial treatment, explanations of test procedures and hospitalization, explanation of medications and usage guidance, venous blood sampling, intravenous injections, securing intravenous lines, placement of urinary catheters, proxy input of medical certificates, and patient transfers [11].

Reducing the working hours of OBGYNs, who often experience overwork, requires understanding the unique characteristics of the medical department regarding OBGYNs, identifying tasks suitable for delegation, and assessing the impact of task shifting on the quality of medical care and working hours. A survey was conducted in 2019 to explore these factors and reported that the “transfer of patients (from operating rooms to the ward)” and “securing the contrast agent line” were the only tasks related to individual work duties that most respondents recognized as having been subject to task shifting, highlighting the limited progress in this area [12]. In Japan, reforms to the way in which doctors work have been carried out since 2018, but the progress of these reforms is unclear.

In contrast, the current research conducted a questionnaire survey among hospital-based OBGYNs across Japan to identify tasks suitable for delegation and gather insights on how task shifting might affect medical care quality and working hours. These results were then compared to the results of the 2019 survey findings to evaluate the progress of task shifting and consider implications for health policy.

Despite task-shifting efforts being promoted globally, the extent of its implementation in OBGYN in Japan remains unclear. Previous studies have primarily focused on general physician workload without specifically assessing how task shifting has progressed in the OBGYN field. This study provides an updated nationwide analysis of task shifting in OBGYNs following Japan’s 2018 physician work reform. By comparing these findings with previous surveys, this study fills a critical gap in understanding the effectiveness and challenges of task shifting in Japan.

Task shifting has been widely implemented in various medical fields worldwide to optimize healthcare resources [1]. In maternal healthcare, studies have shown that delegating specific tasks to mid-level healthcare providers can maintain care quality while reducing physician workload [13]. However, barriers such as professional resistance and regulatory restrictions often hinder progress [14]. In Japan, despite physician work reform efforts initiated in 2018, task shifting in OBGYN remains underdeveloped. This study builds on previous research by assessing the current status and future potential of task shifting in this field.

## 2. Materials and Methods

### 2.1. Subjects

This study employed a cross-sectional survey-based design to assess the progress of task shifting among obstetricians and gynecologists (OBGYNs) in Japan. In total, 1170 hospitals across Japan with OBGYN departments and named in the hospital bed function reporting system were targeted [15], and data were collected via an online questionnaire between 16 November and 20 November 2023, when survey request forms were sent to the managers and physicians of these departments.

The inclusion criteria for this study were the following: (1) licensed OBGYNs currently working in hospital-based settings in Japan and (2) willingness to participate in an online survey. The exclusion criteria included (1) OBGYNs who exclusively worked in private clinics or non-hospital settings and (2) incomplete survey responses.

### 2.2. Measurement and Data Analysis

The survey questionnaire was structured and comprised four main sections: (1) demographic characteristics, (2) perception of task shifting, (3) specific tasks evaluated for task shifting, and (4) perceived impact on workload and quality of care. The questionnaire was pre-tested for validity and reliability, with a Cronbach’s alpha of 0.85, indicating high internal consistency. The survey was distributed via professional medical associations and institutional mailing lists, ensuring broad participation. The questionnaire was administered in Japanese, the native language of the respondents.

Initially, the characteristics of the physicians (sex, age, role, weekly working hours, type of workplace, total hospital bed count, and region) were outlined. Participants were categorized into the following six age groups for consistency across analyses: under 30 years, 30 s < 40 s, 40 s < 50 s, 50 s < 60 s, 60 s < 70 s, and 70 years or older. Workplace type was grouped into four categories: public, national university, private university, and private (excluding private university). Roles were classified as department manager, staff physician, specialized physician, and others. Weekly working hours were segmented into five ranges: less than 40 h, 40–59 h, 60–79 h, 80–99 h, and 100 h or more. The total number of hospital beds was also divided into five groups: <200 beds, 200–399 beds, 400–599 beds, 600–799 beds, and ≥800 beds. In this study, regions were classified based on 344 secondary medical areas (SMAs), which are administrative units used in Japan to organize healthcare services according to population density and medical facility distribution [16].

This study assessed how the respondents felt that their workplace task shifting was progressing (very much progressing, somewhat progressing, not really progressing, hardly progressing, do not know). The dependent variable was subsequently set as physicians who responded with “hardly progressing”, and the explanatory variables were set as the physician attributes (sex, age, occupation, weekly working hours, foundational entity of workplace, total number of hospital beds, and region). These variables were utilized to conduct a multivariate logistic regression analysis to identify the characteristics of physicians who felt that task shifting was hardly progressing in their workplace.

Subsequently, the implementation status of individual task-shifting items was examined, and opinions on their future implementation were gathered (already shifted, should be shifted in the future, should not be shifted in the future, or none of the previous options). For general medical tasks, the Ministry of Health, Labour and Welfare’s Urgent Efforts for Shortening Physicians’ Working Hours [13] was referred to as a reference. For tasks specific to OBGYNs, the same items as those in the 2019 survey were utilized—these items had been created by referencing the opinions of members of the Medical Reform Committee of the Japan Society of Obstetrics and Gynecology, with which the authors of this paper are affiliated [14]. Additionally, we analyzed task-shifting initiatives where opinions varied based on respondent attributes, such as background and future direction.

To ensure the reliability of the survey instrument, Cronbach’s alpha was calculated for key questionnaire sections, yielding a value of 0.85, indicating high internal consistency. Construct validity was assessed through confirmatory factor analysis (CFA), and discriminant validity was established by examining inter-item correlations. Additionally, tests for multicollinearity and common method bias were conducted, with variance inflation factor (VIF) values below 3.0, confirming no significant multicollinearity issues.

Furthermore, a figure was created to illustrate the impact of task shifting on the quality of medical care (greatly improved, somewhat improved, no change, somewhat decreased, greatly decreased, or unknown) and the potential reduction in daily working hours (less than 1 h, 1 to 2 h, 2 to 3 h, 3 to 4 h, 4 h or more, or unknown).

A pilot study was conducted with a small group of 30 OBGYNs to test the clarity and feasibility of the questionnaire. Feedback was used to refine ambiguous questions before full-scale distribution.

For the statistical analysis, *p* values of less than 0.05 were considered significant. All analyses were conducted utilizing STATA 17.0.

### 2.3. Ethical Considerations

This study was conducted in accordance with the principles of the Declaration of Helsinki for research involving human subjects. The study protocol was approved by the Human Research Ethics Committee of Tokyo Healthcare University (approval number: Education 023-03B). Informed consent was obtained from all participants prior to survey participation.

Online-based informed consent was obtained from all the participants. The purpose of this study and the measures to ensure secure data management were outlined on the first page of the questionnaire. Potential participants were informed that their involvement in the study was entirely voluntary. To ensure anonymity and confidentiality, results were analyzed separately from personal information.

## 3. Results

In total, 1170 hospitals with OBGYN departments, identified through Japan’s hospital bed function reporting system, were invited to participate. This study employed a non-probability convenience sampling approach, targeting OBGYNs via hospital administrators and professional networks. A response rate of 36.2% (423 valid responses) was achieved, which represented 16.3% of the 7127 hospital-based OBGYNs in Japan reported in the 2020 Statistics on Physicians, Dentists, and Pharmacists conducted by the Ministry of Health, Labour and Welfare [16]. Table 1 outlines the characteristics of the physicians, including their sex, age, occupation, weekly working hours, workplace foundational entity, total number of hospital beds, and region.

**Figure 1:**
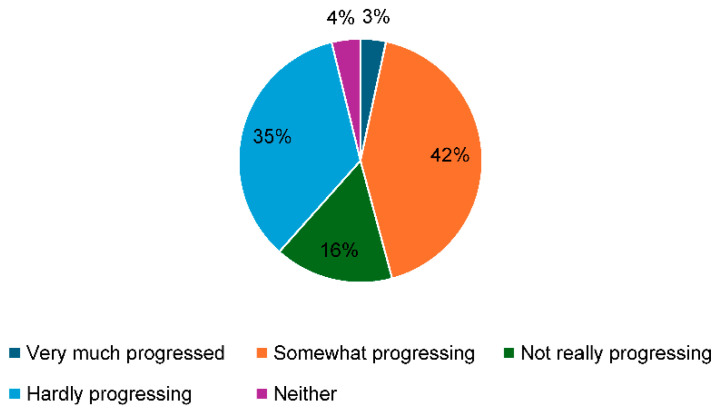
Progress in task shifting in hospitals where participants work.

When asked whether task shifting was progressing in their workplace (Figure 1), 5.9% of participants responded with “very much progressing” and 43.6% with “somewhat progressing”. Conversely, 15.5% responded with “not really progressing”, and 31.2% with “hardly progressing”.

Subsequently, a multivariate logistic regression analysis was conducted on physicians who stated that they believed that task shifting was “hardly progressing”. The explanatory variables were the attributes of the responding physicians (sex, age, occupation, weekly working hours, foundational entity of workplace, total number of hospital beds, and region). We analyzed the characteristics of physicians who felt that progress in task shifting was lacking in their workplaces. The results, shown in Table 2, indicated a significant correlation only with the number of working hours. Utilizing <40 h as a reference, the odds ratios were significantly higher for those working 60 to 79 h at 1.72 (95% CI: 1.06–2.77, *p* = 0.03) and those working over 100 h at 3.50 (95% CI: 1.19–10.25, *p* = 0.02).

Table 3 exhibits the implementation status of individual task-shifting items and opinions on implementation when those items were not yet implemented.

For proxy input tasks, over 70% of respondents indicated “already shifted” or “should be shifted in the future” for all items except “electronic medical record entry”. Over 30% responded “already shifted” for “interviews at first visit (preliminary examinations)” and “preparation of medical certificates and referral letters”, indicating some progress in task shifting. However, 43% of respondents for “electronic medical record entry” felt that it “should not be shifted in the future”. In other countries, it is common to write electric medical records on behalf of physicians, but in Japan, there may be a fixed idea that electric medical records are written by physicians. If there are advanced examples of this practice, such as dictation tools which utilize AI, perhaps the way of thinking about task shifting will change.

Regarding patient explanations and general procedures, over 70% of respondents indicated “already shifted” or “should be shifted in the future” for all items except “utilization of online consultation”. Over 30% responded “already shifted” for “responding to telephone inquiries from patients”, “patient transfer (from operating room to ward, etc.)”, “collection of blood culture specimens”, “securing contrast media lines”, and “securing chemotherapy lines”, indicating some progress in task shifting. For “utilization of online consultation”, 51% of respondents felt it “should not be shifted in the future”, with 43% choosing “none of the previous options”.

Regarding procedures unique to OBGYNs, over 70% of respondents indicated “already shifted” or “should be shifted” for “prescription of routinely used medicines” and “pelvic examination at the time of delivery or water breaking”. Over 30% reported “already shifted” for “pelvic examination at the time of delivery or water breaking” and “adjustment of labor-promoting drugs”, indicating some progress in task shifting. Over 60% indicated that the “prescription of routinely used medicines” should be shifted in the future. Conversely, over 50% felt that “starting labor-promoting drugs for weak labor pains” and “episiotomy and perineal suture” should not be shifted in the future. Opinions were evenly split between “should be shifted in the future” and “should not be shifted in the future” for “fetal screening” and “one-month postpartum examination”.

For OBGYN procedures, the total percentage of “already shifted” and “should be shifted in the future” responses was below 70% for all items. The highest percentages of responses of “should not be shifted in the future” were for “assistant in obstetrics and gynecology surgery”, “intraoperative anesthesia, respiration, and circulation management”, and “postoperative CV removal/PICC insertion”.

**Figure 2:**
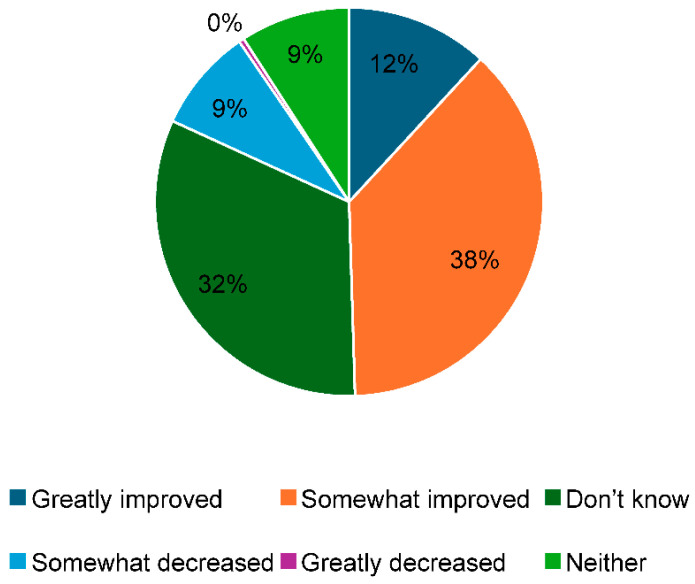
Impact on the quality of medical care if task shifting were to be promoted.

Figure 2 exhibits the impact on the quality of medical care if OBGYNs were to promote task shifting, with 14% responding with “greatly improved”, 47% responding with “somewhat improved”, and 45% responding with “unknown”. Meanwhile, 11% responded with “somewhat decreased”, and 2% responded with “greatly decreased”.

**Figure 3:**
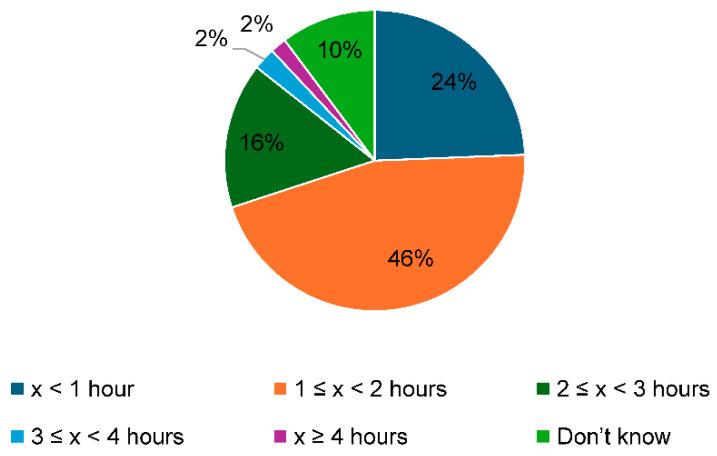
Working hours that could possibly be reduced by task shifting.

Figure 3 exhibits the working hours per day that OBGYNs believe could be shortened with task shifting. The highest number of responses (46%) indicated “1 ≤ x < 2 h”, followed by 24% for “x < 1 h”, and 16% for “2 ≤ x < 3 h”. The responses “3 ≤ x < 4 h” and “x ≥ 4 h” each accounted for 2% of the total.

## 4. Discussion

### 4.1. Progress of Task Shifting and Related Factors

In this study’s questionnaire of OBGYNs working at obstetric and gynecology hospitals across Japan, when respondents were asked about the progress of task shifting in their workplaces, 49.6% responded with either “very much progressing” or “somewhat progressing”, while 31.7% responded with “hardly progressing”. Possible factors contributing to this disparity in perception among physicians include variations in the progress of physicians’ work style reforms across medical institutions and disparities in opinion regarding the extent of the implementation of task shifting in OBGYNs’ work. In our 2019 survey, 34.6% responded with “hardly progressing”. Although the current values have slightly decreased compared to this prior value, the results still indicate that numerous OBGYNs perceive minimal progress in task shifting [14].

Regarding the characteristics of physicians who perceived task shifting as “hardly progressing”, no significant correlations were found with sex, age, occupation, foundational entity of workplace, total number of hospital beds, or region. However, significant correlations were found with weekly working hours of 60–79 h (20.6% of respondents) and 100 h or more (1.5% of respondents). Working 60 h per week equates to an additional 4 h of overtime per day compared to the standard legal working hours of 8 h per day and 40 h per week, as outlined in the Labor Standards Act. This can result in overtime hours surpassing approximately 80 h per month. Such extended working hours are often termed the “overwork death threshold”, as they are closely linked to an increased risk of mental health disorders and cardiovascular diseases as a result of psychological stress, meeting the certification criteria for occupational hazards [17,18,19,20,21]. While various factors contribute to long working hours, priority should be given to shortening the working hours of OBGYNs who work such extended hours by promoting task shifting.

### 4.2. Status of Each Item Related to Task Shifting

Table 3 illustrates task shifting from physicians to non-physician occupations in OBGYN care in Japan. Among these, only “patient transfer (from operating room to ward, etc.)”, “collection of blood culture specimens”, and “securing chemotherapy lines” had over 50% of OBGYNs respond as “already shifted”, highlighting the current lack of progress in task shifting. Progress in task shifting was more evident for “proxy input” and “patient explanations and general procedures” compared to “procedures unique to obstetrics and gynecology”. These areas have also been identified as targets for task shifting even in the Urgent Efforts for Shortening Physicians’ Working Hours announced by the Ministry of Health, Labour and Welfare in 2018. Task shifting is expected to further progress in the future through the reform of the work style of government-led physicians [1]. However, nearly half of the respondents expressed opposition to task shifting, particularly with regard to “electronic medical record entry” (proxy input). In other countries, physicians commonly record what they write in their medical records utilizing a voice recorder, and clerical staff then input the information on the physicians’ behalf; therefore, the factors behind why such efforts have not progressed in Japan, including reasons for why physicians oppose such efforts, need to be investigated further in the future.

Efforts to implement task shifting for specific procedures in obstetrics and gynecology have been actively pursued in low-,middle-, and high-income countries. Studies have highlighted the role of task shifting in optimizing the use of medical resources [13,14,22,23]. The WHO’s recommendations [7] on task shifting in maternal and newborn healthcare emphasize that tasks such as the “adjustment of labor-inducing drugs”, “bimanual uterine compression”, and “episiotomy and perineal suturing”, which were included in the questionnaire of this study, should be delegated from physicians to auxiliary nurse midwives. Regarding these items, in this study, the percentage of responses reflecting “already shifted” exceeded 30% for “adjustment of labor-promoting drugs”, indicating a certain degree of task-shifting progress, whereas the percentage of responses reflecting “should not be shifted in the future” was highest for “episiotomy and perineal suture” and “bimanual compression of the uterus”, confirming that there were numerous cautious opinions regarding task shifting. Priority should be given to items with a high percentage of “should be shifted in the future” responses when considering task-shifting initiatives.

Comparing the 2019 survey results, items that exhibited a change of ≥20% included increases in “already shifted” for “securing chemotherapy lines” and “should be shifted in the future” for “fetal ultrasounds in prenatal checkups” and “prescription of routinely used drugs”, indicating a certain degree of progress in task shifting; meanwhile, increases in “should not be shifted in the future” for “starting labor-promoting drugs for weak labor pains”, “episiotomy and perineal suture”, “assistant in obstetrics and gynecology surgery”, and “intraoperative anesthesia, respiration, and circulation management” indicate regression in perceptions toward task shifting.

Simultaneously educating and certifying the midwives and nurses who would receive the shifted task is a possible option for task-shifting procedures specific to OBGYNs [7]. In Japan, the Japanese Midwives Association established the advanced midwifery system, which is utilized for assessing and certifying that an individual’s practical abilities in midwifery have reached a certain level [24]. Additionally, the Japanese Nursing Association has considered establishing a nurse practitioner (tentative name) system, but Japan does not have a qualification system for nurse practitioners who are recognized in the United States as well as in other countries and who are allowed to conduct a certain level of diagnosis and treatment without receiving instructions from a physician [25]. Promoting task shifting requires the development of a qualification system and the necessary legislation in order to delegate the tasks that are currently conducted by OBGYNs to qualified staff, such as advanced midwives or the Japanese version of nurse practitioners.

### 4.3. Impact of Promoting Task Shifting on the Quality of Medical Care

Regarding how OBGYNs’ promotion of task shifting may impact the quality of medical care, the majority of OBGYNs responded that the quality would improve, and only 13% responded that the quality would decrease. A decrease in the quality of medical care as a result of task shifting should be avoided, but the results clarified that at least the majority of the OBGYNs who responded to this survey did not feel this way.

Regarding the number of working hours per day that OBGYNs believed could be shortened by task shifting, “1 to 2 h” had the highest number of responses at 45%, while 30% of respondents notably replied “<1 h.” As mentioned earlier, OBGYNs are said to have some of the most extended working hours among all clinical physicians, with reports exhibiting that 20.5% of OBGYNs have average working hours of ≥80 h weekly [10]. In this study, 5.5% of physicians responded that their working hours were ≥80 h weekly. For such physicians, even if their daily working hours were reduced by two hours as a result of task shifting, they would still be at a level that exceeds that of death resulting from overworking. There is a need for further measures to shorten working hours in addition to task shifting, such as strengthening the physician system by consolidating delivery medical institutions [26].

This study has significant implications for both healthcare policy and clinical practice. From a managerial perspective, promoting task shifting can help alleviate physician burnout and improve work–life balance, particularly in high-demand specialties such as OBGYN. Socially, implementing structured task-shifting policies can enhance healthcare access and efficiency without compromising patient safety. Future policy efforts should focus on establishing clear task delegation guidelines and providing training programs for mid-level healthcare professionals.

### 4.4. Limitations

This study has several limitations. First, the response rate was 36.2%, which may have introduced selection bias. Second, as the survey was conducted exclusively among hospital-based OBGYNs, findings may not be generalizable to private clinics. Additionally, the study did not assess the perspectives of the midwives or nurses who would be involved in task shifting, which is crucial for understanding implementation feasibility. Future research should incorporate a broader range of stakeholders to provide a more comprehensive evaluation of task shifting.

## 5. Conclusions

In conclusion, this study highlights the limited progress of task shifting in Japanese OBGYN departments despite physician work reform efforts. While task shifting has potential benefits in reducing physician workload and improving efficiency, resistance remains, particularly for tasks requiring direct physician oversight. Future policy efforts should focus on establishing standardized guidelines, improving training for non-physician staff, and fostering institutional support to facilitate sustainable task delegation.

## Figures and Tables

**Table 1:** Respondent attributes.

No. of Respondents	1164	
Sex		
Female	580	49.8%
Male	584	50.2%
Age		
Under 30 years	130	11.2%
30 s < 40 s	374	32.1%
40 s < 50 s	318	27.3%
50 s < 60 s	187	16.1%
60 s < 70 s	141	12.1%
70 years or older	14	1.2%
Occupation		
Department manager	325	27.9%
Staff	582	50.0%
Specialized physician	223	19.2%
Other	34	2.9%
Working Hours per Week		
Less than 40 h per week	148	12.7%
40–59 h per week	710	61.0%
60–79 h per week	240	20.6%
80–99 h per week	46	4.0%
100 h or more per week	17	1.5%
Foundational Entity of Employer		
Public	668	57.4%
National university	121	10.4%
Private university	69	5.9%
Private	306	26.3%
Employer’s Total No. of Beds		
Less than 200 beds	90	7.7%
200–399 beds	320	27.5%
400–599 beds	399	34.3%
600–799 beds	203	17.4%
800 or more beds	152	13.1%
Employer’s Regional Classification		
Urban	558	47.9%
Intermediate	489	42.0%
Rural	117	10.1%

**Table 2:** Association between task shifting and physician characteristics.

	OR	95% CI	*p*-Value
Sex			
Female	Reference		
Male	1.11	0.84–1.47	0.46
Age			
Under 30 years	Reference		
30 s < 40 s	0.98	0.56–1.73	0.95
40 s < 50 s	0.77	0.41–1.44	0.41
50 s < 60 s	0.55	0.27–1.12	0.10
60 s < 70 s	0.83	0.39–1.77	0.64
70 years or older	0.64	0.15–2.77	0.15
Occupation			
Department manager	Reference		
Staff	0.95	0.63–1.41	0.79
Specialized physician	0.75	0.42–1.36	0.35
Other	1.06	0.45–2.48	0.90
Working Hours per Week			
Less than 40 h per week	Reference		
40–59 h per week	1.20	0.79–1.84	0.39
60–79 h per week	1.72	1.06–2.77	0.03 *
80–99 h per week	1.48	0.70–3.11	0.30
100 h or more per week	3.50	1.19–10.25	0.02 *
Foundational Entity of Employer			
Public	Reference		
National university	1.48	0.90–2.45	0.13
Private university	0.97	0.48–1.97	0.94
Private	0.85	0.60–1.20	0.35
Employer’s Total No. of Beds			
Less than 200 beds	Reference		
200–399 beds	1.14	0.63–2.06	0.67
400–599 beds	1.23	0.67–2.24	0.50
600–799 beds	1.49	0.76–2.90	0.25
800 or more beds	2.01	0.95–4.24	0.07
Workplace			
Urban	Reference		
Intermediate	0.96	0.73–1.27	0.78
Rural	1.00	0.62–1.61	1.00

* *p* < 0.05.

**Table 3:** Thoughts toward implementation of task shifting.

1. Proxy B4:F38				
	**Already shifted**	**Should be shifted in the future**	**Should not be shifted in the future**	**None of the previous options**
“Interviews at first visit (preliminary examinations)”	480	375	165	144
Ordering tests, prescriptions, treatments, etc.	205	611	186	162
Hospitalization/surgery reservation	171	701	165	127
Preparation of medical certificates and referral letters	382	546	129	107
Preparation of discharge summary	178	702	171	113
Electronic medical record entry	69	407	497	191
Case registration (cancer registration, etc.)	187	819	50	108
2. Patient explanations and general procedures				
	**Already shifted**	**Should be shifted in the future**	**Should not be shifted in the future**	**None of the previous options**
Responding to telephone inquiries from patients	447	473	97	147
Explanations using pamphlets and video	252	720	73	119
Utilization of online consultation	27	592	181	364
Patient transfer (from operating room to ward, etc.)	598	432	62	72
Collection of blood culture specimens	661	412	30	61
Securing contrast media lines	419	550	63	132
Securing chemotherapy lines	595	438	59	72
3. Procedures unique to obstetrics and gynecology				
	**Already shifted**	**Should be shifted in the future**	**Should not be shifted in the future**	**None of the previous options**
Fetal ultrasounds in prenatal checkups	83	642	237	202
Fetal screening	71	480	432	181
Prescription of routinely used medicines	50	759	207	148
Pelvic examination at the time of delivery or water breaking	440	502	118	104
Starting labor-promoting drugs for weak labor pains	62	320	640	142
Adjustment of labor-promoting drugs	433	355	256	120
Episiotomy and perineal suture	14	279	688	183
Bimanual compression of the uterus	48	376	531	209
One-month postpartum examination	25	501	453	185
4. Procedures in obstetrics and gynecology				
	**Already shifted**	**Should be shifted in the future**	**Should not be shifted in the future**	**None of the previous options**
Assistant in obstetrics and gynecology surgery	23	286	636	219
Intraoperative anesthesia, respiration, and circulation management	110	300	549	205
Postoperative drain management/removal	30	473	467	194
Postoperative CV removal/PICC insertion	42	412	497	213
Postoperative wound management (cleaning, suture removal, hook removal)	41	503	431	189

## Data Availability

The data supporting the findings cannot be shared publicly for ethical reasons.
